# Oxidative Effects during Irreversible Electroporation of Melanoma Cells—In Vitro Study

**DOI:** 10.3390/molecules26010154

**Published:** 2020-12-31

**Authors:** Wojciech Szlasa, Aleksander Kiełbik, Anna Szewczyk, Nina Rembiałkowska, Vitalij Novickij, Mounir Tarek, Jolanta Saczko, Julita Kulbacka

**Affiliations:** 1 Faculty of Medicine, Wroclaw Medical University, 50-367 Wroclaw, Poland; Wojciech.Szlasa@outlook.com (W.S.); Aleksander.Kielbik@outlook.com (A.K.); 2 Department of Molecular and Cellular Biology, Faculty of Pharmacy, Wroclaw Medical University, 50-556 Wroclaw, Poland; A.Szewczyk@umed.wroc.pl (A.S.); Nina.Rembialkowska@umed.wroc.pl (N.R.); Jolanta.Saczko@umed.wroc.pl (J.S.); 3 Department of Animal Developmental Biology, Institute of Experimental Biology, University of Wroclaw, 50-328 Wroclaw, Poland; 4 Faculty of Electronics, Vilnius Gediminas Technical University, 03227 Vilnius, Lithuania; Vitalij.Novickij@vgtu.lt; 5 Université de Lorraine, CNRS, LPCT, F-54000 Nancy, France; Mounir.Tarek@univ-lorraine.fr

**Keywords:** IRE, oxidative stress, melanoma, permeabilization, membrane alternations

## Abstract

Irreversible electroporation (IRE) is today used as an alternative to surgery for the excision of cancer lesions. This study aimed to investigate the oxidative and cytotoxic effects the cells undergo during irreversible electroporation using IRE protocols. To do so, we used IRE-inducing pulsed electric fields (PEFs) (eight pulses of 0.1 ms duration and 2–4 kV/cm intensity) and compared their effects to those of PEFs of intensities below the electroporation threshold (eight pulses, 0.1 ms, 0.2–0.4 kV/cm) and the PEFs involving elongated pulses (eight pulses, 10 ms, 0.2–0.4 kV/cm). Next, to follow the morphology of the melanoma cell membranes after treatment with the PEFs, we analyzed the permeability and integrity of their membranes and analyzed the radical oxygen species (ROS) bursts and the membrane lipids’ oxidation. Our data showed that IRE-induced high cytotoxic effect is associated both with irreversible cell membrane disruption and ROS-associated oxidation, which is occurrent also in the low electric field range. It was shown that the viability of melanoma cells characterized by similar ROS content and lipid membrane oxidation after PEF treatment depends on the integrity of the membrane system. Namely, when the effects of the PEF on the membrane are reversible, aside from the high level of ROS and membrane oxidation, the cell does not undergo cell death.

## 1. Introduction

Ablation techniques are commonly used in medicine for the removal of diseased (especially cancerous) tissues [1,2]. The process involves the application of various damage-causing methods to induce instant and focused necrosis in the region of interest [3,4,5,6]. Ablation might be divided into chemoablation and physical ablation [7,8,9]. The former involves, for instance, the use of chemicals, e.g., ethanol, hypertonic saline, or acetic acid directly to the tissue [10]. Physical ablation relates to physical methods inducing damage to treated tissues and includes cryoablation, laser ablation, radiofrequency ablation, microwave ablation and fulguration [3]. Cryoablation is used in surgical oncology for small prostate tumors, renal cell carcinoma and breast cancer treatments [11,12,13] as a treatment for cardiac ablation for patients with arrhythmias [14,15] and in dermatology, where it is used for rejuvenation of aged skin and removal of skin spots and lesions [16,17]. Conversely, laser ablation induces damage by high-temperature-aided vaporization of the tissue [18,19]. Laser scalpels are widely used in surgery due to their high effectivity in soft and hard tissue removal and blood-vessel-closing capabilities [20,21]. Radiofrequency ablation is a minimally invasive procedure for tissue removal, especially used in hard tissue disorders [22]. Microwave ablation differs from the latter by the frequency of the applied waves—300 MHz-300 GHz and 450–500 kHz, respectively [23,24]. The last of the commonly used ablation methods is fulguration (electric surgery), which involves heat generation following the application of high-voltage electric fields to, for instance, superficial tissues and urological tumors [25,26]. The heat generated by the electric current flow induces necrotic cell death focally in the region reached by the catheter [27].

Melanoma treatment directives include the surgical removal of the tumor, advocating for the search for new ablation methods [28]. Although the standard ablation techniques cited above are effective, new methods that induce necrosis are currently under active research. Among the promising ones, the application of electric pulses to target tissues has emerged recently as a viable and effective alternative technique [29]. The method differs from fulguration by the fact that heat is not used on the effector of the target cells [1]. The molecular mechanisms at play involve rather the generation of damage to the cell membranes leading to leakage or permeability thought to stem from pore formation [30]. In this technique, called often, therefore, electroporation, when low-voltage pulses are applied, the increased permeability of the cell membranes is transient, and after a definite time lapse, the cells recover their integrity. During this time lapse, the inflow of compounds to the cytoplasm may take place, which is used today as an effective way for drug or gene delivery [31,32]. In contrast, when rather high-voltage pulses are applied, the membranes might be permanently damaged, in which case, the concerned cells undergo necrosis [33]. The technique is termed, in this case, irreversible electroporation (IRE). The early cell death process is characterized by the specific bubbling of the plasmalemma, by which the cells release the organelles and genetic material to the extracellular space [34]. Interestingly, as IRE acts mainly on the permeability of the cell membranes, it is considered safe for the treatment of tumors located near critical structures. Moreover, when the targeted area to treat is near large blood vessels, IRE prevents the so-called “heat sink effect” which, in the case of thermal ablation methods, might lead to incomplete ablation [35]. Today, IRE has been proved effective in clinical settings for prostate and renal cancer treatments [36], hepatocellular cancer treatment [37] and pancreatic cancer ablation [29].

Rubinsky et al. proposed that the cytotoxic effect of the electroablation is caused by at least three effects: irreversible electroporation without electrolysis, irreversible electroporation combined with electrolysis and reversible electroporation combined with electrolysis [38]. Electrolysis is responsible for the generation of radical oxygen species (ROS) during the redox reactions occurring on the surface of the electrodes. In this case, ROS are localized in the extracellular electroporation buffer and may affect the cell plasma membranes. However, the application of pulsed electric fields (PEFs) could also be responsible for the generation of the inner-cell derived ROS. Indeed, sub-microsecond pulses induce mitochondria damage and release of ROS to the cytoplasm [39]. The excessive amount of ROS created can attack lipids, proteins and DNA. Because of their structure and location, lipids are vulnerable molecules in regard to oxidative attack. In particular, oxidative damage to cell membranes causes lipid peroxidation, which is a reaction that takes place through a chain reaction mechanism and that typically affects unsaturated lipids.

As reported in a recent review [30], several studies demonstrated over two decades ago that microsecond and millisecond electric pulses induce the generation of reactive oxygen species (ROS) and consequently oxidative damage of unsaturated lipids in cell membranes. Data show that ROS concentration and the extent of lipid peroxidation increases with electric field intensity, pulse duration and the number of pulses and is correlated with cell membrane permeability, membrane resealing time and cell damage [40,41,42,43,44]. Various studies proved the relation between electropermeabilization and the oxidation of membrane lipids as discussed in [40,45].

Today, however, the role of cell membrane oxidation, its impact on cell permeability and viability related to PEF treatments remain not fully understood. Based on the current knowledge, it could be assumed that IRE might induce cell death in an oxidation-related pathway. Therefore, we performed the study to better characterize the relation between ROS, lipid oxidation, membrane permeability and cell death after IRE.

This study aimed to analyze if IRE can induce the oxidative effects in melanoma (A375) cells subject after the application to IRE protocol pulses. In addition, we aimed to compare IRE protocol with pulses of intensity below the electroporation thresholds, i.e., pulses that do not induce plasma membrane permeabilization but that may generate ROS in the electrolytic pathway. The so-called microsecond IRE protocol (2–4 kV/cm, 0.1 ms pulse duration, eight pulses) was applied to induce cell death among the melanoma cells. To analyze the oxidative stress generation by electric fields below the electroporation threshold, we applied PEFs of intensity 0.2–0.4 kV/cm, 0.1 ms pulse duration (eight pulses), then we increased pulse duration up to 10 ms to further increase the electrolysis-related effect on the melanoma cells. First, the cell membrane permeability was analyzed with Yo-Pro uptake flow cytometry studies. Further, we analyzed the cell membrane permeabilization kinetics following the application of the PEF treatments by trypan blue staining. The long-term viability of the cells was measured by MTT viability assay, 24 h after application of the PEFs. The ultrastructure of the cells was analyzed with holotomography microscopy. Due to the impact of the PEF on the biomembranes and the nuclear content fragmentation, the morphological changes in the organization of the membrane system, as well as the nuclear content, were assessed by staining of the membranes with CellMask Deep Red and the nuclei with DAPI. Finally, the generation of ROS and the subsequent oxidation of the lipids were assessed to analyze the oxidative effect of the three PEF protocols used.

## 2. Results

### 2.1. Yo-Pro Uptake Studies

**Figure 1:**
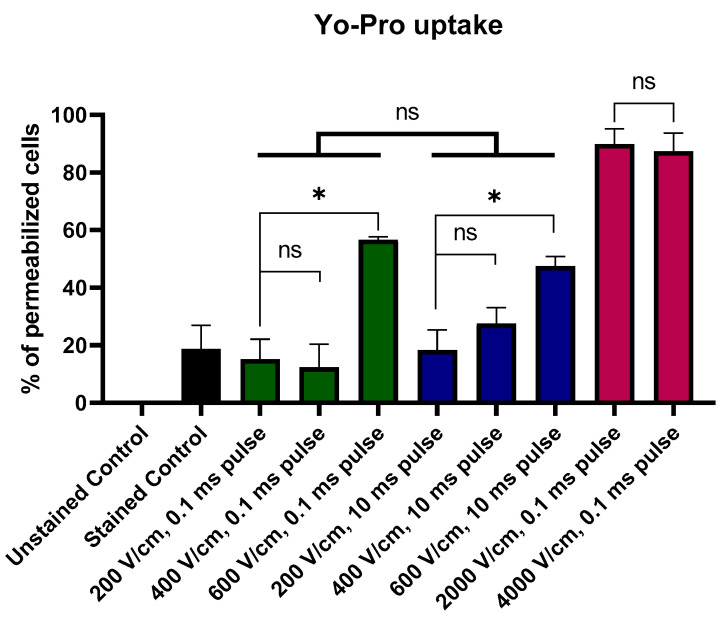
Permeability of melanoma cells as reported by the Yo-Pro uptake flow cytometry studies. Note that irreversible electroporation (IRE) protocols induce high-level permeabilization. Pulsed electric fields (PEFs) of lower intensities induce pulse-duration-dependent permeabilization. Black Bar represents control; green bars represent 200–600 V/cm, 0.1 ms pulses; blue bars represent 200–600 V/cm, 10 ms pulses; red bars represent 2–4 kV/cm, 0.1 ms pulses. Data presented as average ± SD. One-way ANOVA: * *p* < 0.05, ns *p* > 0.05.

Figure 1 presents the uptake of Yo-Pro following the PEF treatment. Melanoma cells were suspended in the electroporation buffer with Yo-Pro and instantly treated with the electric field. The cells were then incubated with the stain for 1 min and analyzed with flow cytometry. The study showed the changes in membrane permeability as a function of the applied electric field. There were no differences between the samples treated with 200–600 V/cm 0.1 and 10 ms pulses. Cell membrane permeability did not increase in response to the elongation of the electric pulse. In addition, there were no statistical differences between cells treated with 200 and 400 V/cm PEFs, and the first significant increase in the Yo-Pro uptake was observed following the application of the 600 V/cm PEF, thus this value was further considered as the electroporation threshold. Melanoma cells treated with IRE protocols were maximally saturated with Yo-Pro and no difference between 2 and 4 kV/cm PEFs was observed in the dye uptake.

### 2.2. Trypan Blue Staining of Permeabilized Cells

**Figure 2:**
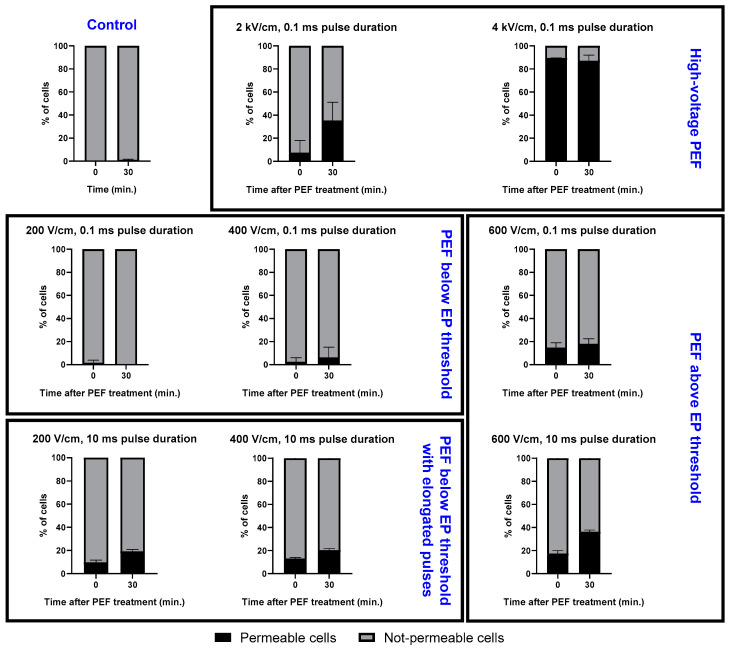
Trypan blue staining shows pore resealing after 30 min. PEFs below the EP threshold induce the pulse-duration-dependent permeabilization for the stain. Data presented as the % of cells ± SD.

To analyze the kinetics of membrane permeability in time, trypan blue stain uptake studies were performed. After the application of PEFs, the cells were resuspended in trypan blue solution and monitored in time. This attempt allowed for the assessment of pore stability and reversibility of the electroporation. Figure 2 reports the trypan blue staining studies of the A375 cell line after the application of PEFs. The study proved the potency of IRE protocols in irreversibly permeabilizing the A375 melanoma cell line, in particular the drastic instantaneous effect induced by the 4 kV/cm pulses. We note as well that for the 2 kV/cm PEFs, the ratio of cells that are permeable increases gradually with time. The middle panel of Figure 2 shows that the application of the (200–600 V/cm, 0.1 ms) PEF leads to a notable increase in the number of instantly permeabilized cells with the increase in PEF intensity and only a smaller subsequent uptake of the stain over time. Permeabilization of the cell membrane is also present in the necrotic cells. In contrast, the long (200–600 V/cm, 10 ms) pulses led to a measurable increase of the permeability to trypan blue in the first 30 min after their application.

Therefore, interestingly, the protocol involving the application of the 10 ms pulses led to a substantial increase in the fraction of permeable cells’ ratio over time. Most notably, our data showed unexpectedly that a PEF of 600 V/cm (10 ms) can induce as much permeabilization as the 2 kV/cm IRE PEFs. Owing to the fact that trypan blue stains not only necrotic but all permeabilized cells as their membrane integrity is interrupted, we conducted an additional experiment to better assess the death-inducing effects of the PEF protocols considered.

### 2.3. Viability Assay

**Figure 3:**
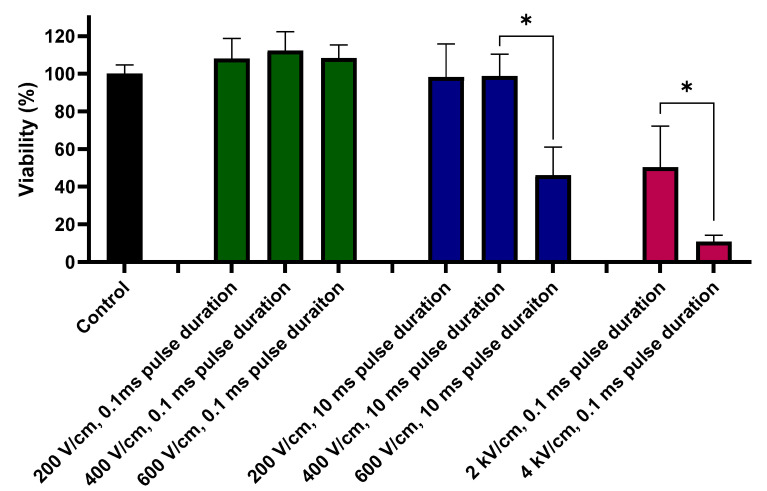
Viability (%) of the cells after standard IRE protocol (2 and 4 kV/cm, 0.1 ms pulse duration, 8 pulses), 200–600 V/cm, 0.1 ms pulse duration, 8 pulses PEFs and PEFs with the elongated pulse duration (200–600 V/cm, 10 ms pulse duration, 8 pulses). Green bars represent 200–600 V/cm, 0.1 ms pulses; blue bars represent 200–600 V/cm, 10 ms pulses; red bars represent 2–4 kV/cm, 0.1 ms pulses. Data presented as average ± SD. One-way ANOVA test analysis: * *p* < 0.0001.

Figure 3 reports the viability of the A375 cells 24 h after the application of the three types of PEFs. The mitochondrial activity assay (MTT) results provide very interesting additional information to interpret the results from trypan blue staining studies. Namely, the most extensive cytotoxic effect of the PEF treatments was observed following the application of the high-voltage IRE (2–4 kV/cm, 0.1 ms) protocols. In these instances, the cells’ viability decreased to ~50% when treated with the 2 kV/cm pulses and to a remarkable ~10% when treated with the 4 kV/cm pulses.

For the 200–600 V/cm PEFs range, a substantial decrease in the cell viability was observed only when the cells were subject to the long, 10 ms pulses of 600 V/cm.

For the purpose of this study, further experiments considered only the IRE protocol. PEFs of electric field intensities (200 and 400 V/cm both 0.1 and 10 ms pulses), i.e., below the EP threshold, were used to assess the cellular mechanism of the oxidative and cytotoxic effects the cells undergo during IRE.

### 2.4. Nuclei and Membrane Staining

**Figure 4:**
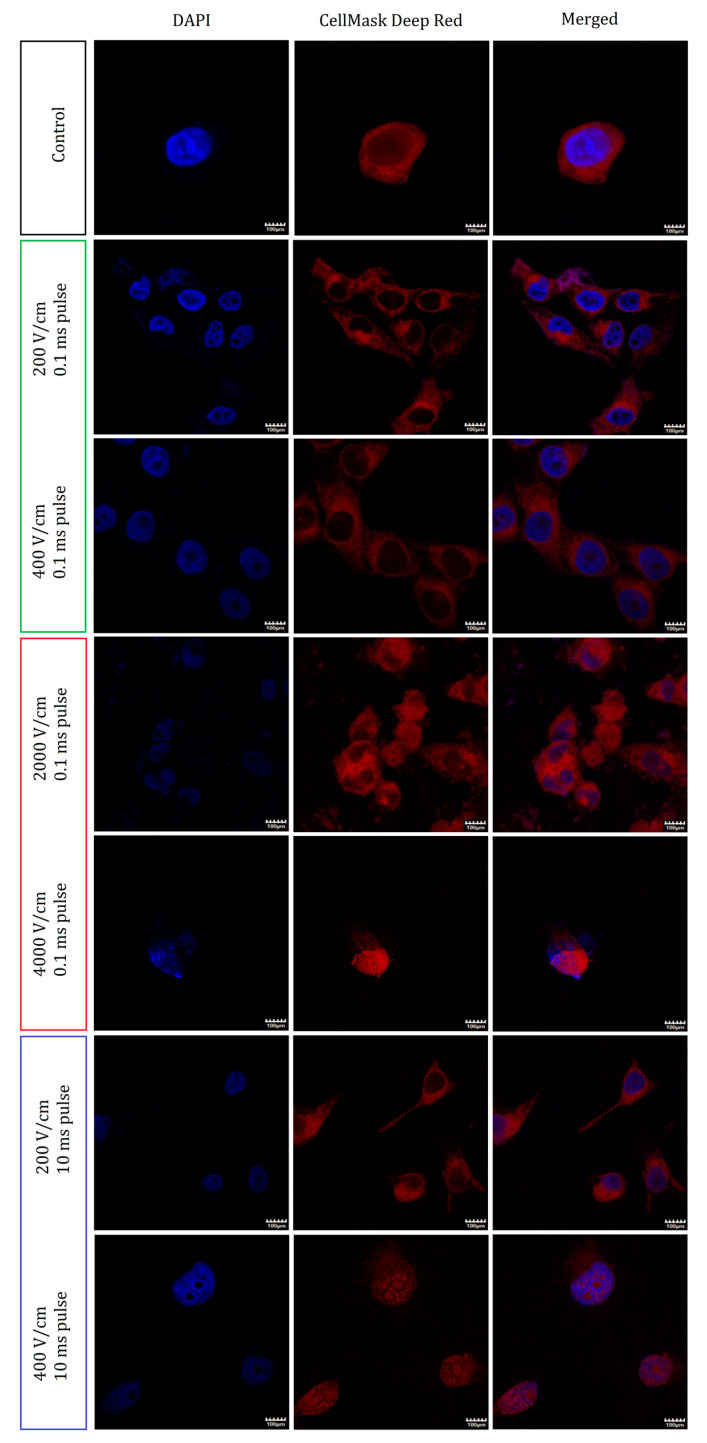
Immunofluorescence staining studies of cells subject to the PEF treatments. The two left columns report the standalone fluorescence of the nuclei and of the cell membranes. On the merged photographs (right panel), both channels are presented together. Each row represents a different electroporation protocol as indicated. The first two present the protocol involving the application of the pulses below EP threshold (200, 400 V/cm, 0.1 ms pulse duration, 8 pulses), next two the increased electric field intensity (IRE, 2–4 kV/cm, 0.1 ms pulse duration, 8 pulses) and the last two the increased pulse duration (200–400 V/cm, 10 ms pulse duration, 8 pulses). Scale bar—100 μm.

To determine the origins of the viability loss of the cells, we studied the morphological changes in the organization of the membranes of the A375 cells. Figure 4 reports the confocal microscopy staining studies of the cells and their nuclei following the treatment with the various PEF protocols. When subject to fields below the electroporation threshold (200–400 V/cm, 0.1 ms), the data show subtle changes in the structure of the cells. With increasing PEF intensity (200–400 V/cm), the cells appear to become spherical and lose their spindle-like shape. In general, however, the cells retained their integrity and no cell aggregation was observed.

A ten-fold increase in the applied electric field intensity, i.e., under IRE conditions, a high level of membrane and nuclei fragmentation was observed. Namely, for the 2 kV/cm PEF treatment, small structures stained with DAPI and CellMask were observed outside the cells. Furthermore, cell-size membranous structures that lack nuclei were also formed. The cells aggregated and no borders were observed between them. Finally, when increasing the electric field intensity from 2 to 4 kV/cm, the cell’s morphology was significantly disrupted: the later shrunk and became spherical.

When subject to the longer 10 ms PEFs instead of the more intense ones, the A375 cells reduced in size and lost their typical elongated morphology. However, their shapes remained elongated and slim after the application of the low-intensity 200 V/cm pulses. When subject to the higher (400 V/cm) intensity PEFs, the cells changed the relative organization of the membrane and nuclear content and thus became dismembered.

### 2.5. Holotomographic Microscopy Studies

**Figure 5:**
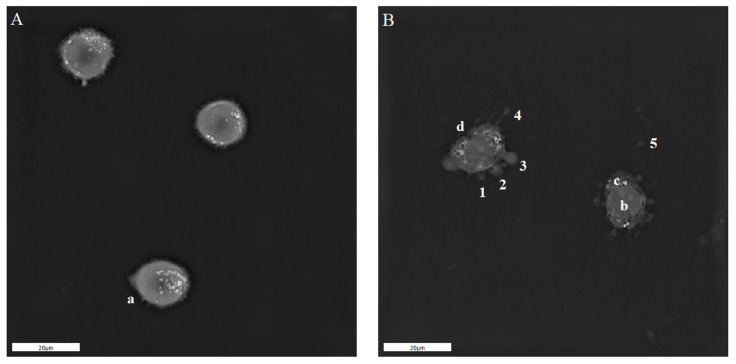
Holotomographic microscopy studies of A375 cells after 4 kV/cm, 8 pulses, 0.1 ms pulse duration treatment. Photograph on the left side shows the normal morphology and filopodia of the cells suspended in the EP buffer (**A**). Photograph on the right side of the figure (**B**) presents blebbing cells after 4 kV/cm, 0.1 ms PEF treatment. 1, 2, 3—evolution of the blebs, 4—bleb release from the membrane, 5—free bleb outside the cell; a—filopodia, b—nucleus, c—cytoplasm, d—membrane invagination; scale bar—20 μm.

The previous data indicated that the application of PEFs using IRE protocols to melanoma cells induces blebbing and releases membranous structures containing genetic material to the extracellular environment. To further characterize the origin and the mechanism of the formation of such defects, we used holotomographic microscopy (see Figure 5). The samples exhibited extensive bubbling of the A375 cells, immediately following the application of the 4 kV/cm PEFs. The cells, therefore, released their cytoplasmic content to the extracellular space. During this process, bubbles were continuously formed. Accordingly, such a process might be considered time-dependent.

### 2.6. ROS Cellular Content Staining Studies after PEF Treatment

**Figure 6:**
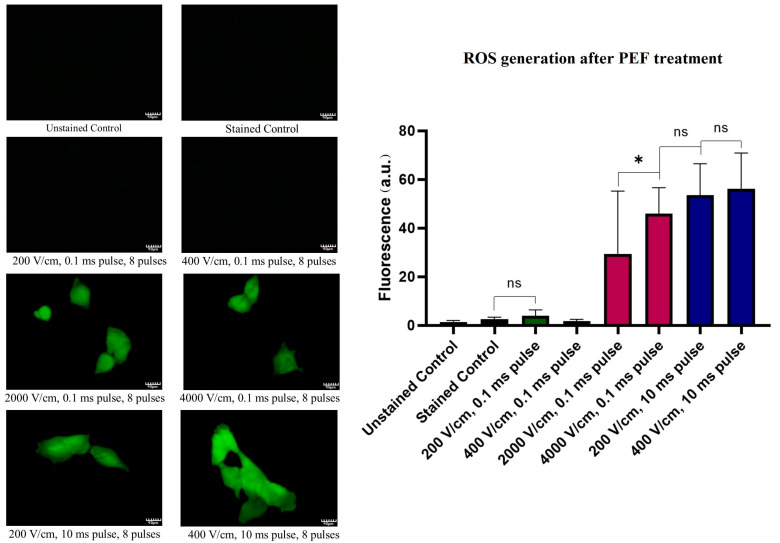
Oxidation stress induction 12 h after PEF treatment. The left side panel reports representative fluorescent photographs (40×) of the cells stained with CM-H_2_DCFDA. The right panel reports the statistical analysis of the samples. Green bars represent 200–400 V/cm, 0.1 ms pulses; blue bars represent 200–400 V/cm, 10 ms pulses; red bars represent 2–4 kV/cm, 0.1 ms pulses. The fluorescence of the samples presented as the mean ± SD. One-way ANOVA analysis: * p < 0.05, ns *p* > 0.05. Scale bar—40 μm.

Figure 6 allows one to follow the amount of ROS production in melanoma cells following the PEF treatments. The ROS content in the cell might arise from both the extracellular space (i.e., electrolysis) or the release from the cellular organelles (i.e., mitochondria leakage). The application of the 0.1 ms pulses of intensities below the electroporation threshold does not seem to induce an increase in oxidative stress. However, the application of the IRE PEFs protocols (10-fold higher intensity pulses), leads to a substantial increase of the cell’s fluorescence indicating an increase of ROS production. Remarkably, when the 10 ms pulses are applied, the ROS content reaches the highest values among all tested PEF protocols. In this case, there was no statistically significant difference between the cells treated with 200 or 400 V/cm fields.

### 2.7. Lipid Oxidation

**Figure 7:**
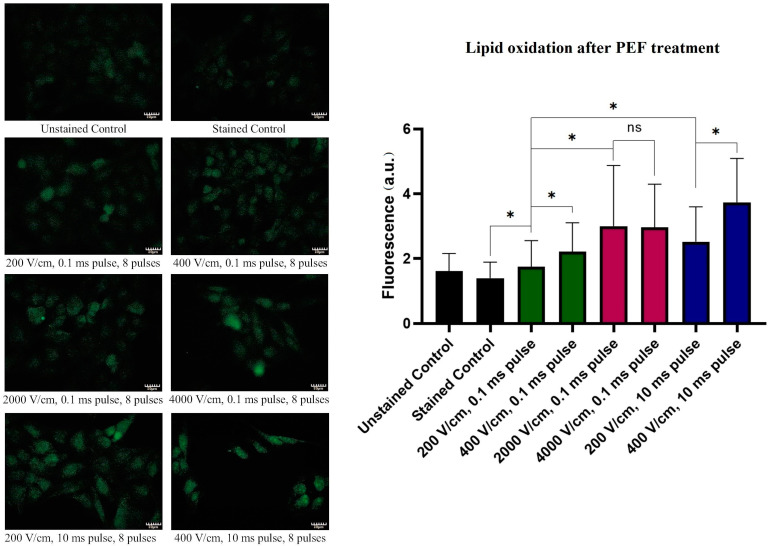
Lipid oxidation induction 36 h after electroporation. The left panel depicts representative fluorescent photographs of the cells stained with the Click-iT^TM^ kit. On the right panel, the statistical analysis of the samples is provided. Green bars represent 200–400 V/cm, 0.1 ms pulses; blue bars represent 200–400 V/cm, 10 ms pulses; red bars represent 2–4 kV/cm, 0.1 ms pulses. The fluorescence is presented as the mean ± SD. One-way ANOVA analysis: * *p* < 0.05, ns *p* > 0.05. Scale bar—80 μm.

Figure 7 reports the changes in the A375 cells’ lipid oxidation following treatments with PEFs. Similarly to the ROS-stress induction studies, the application of the 200–400 V/cm, 10 ms electric pulses induced the highest oxidation of the lipids. For such pulses, the oxidation increased gradually with increasing electric field intensity. Interestingly, no significant difference was observed between samples electroporated with the IRE PEF protocols (0.1 ms pulse duration, eight pulses) at 2 and 4 kV/cm.

## 3. Discussion

The results presented herein show the potency of PEF in vitro treatments of human melanotic melanoma cells (A375), using standard IRE protocols (2–4 kV/cm, 0.1 ms, eight pulses). We attempted to shed light on the process under which such PEF treatments induce cell death. Previously, several in vitro attempts on other cell lines proved the effectiveness of this treatment modality [46,47]. The study of Miller et al. highlighted the fact that the cytotoxic effect of IRE is complex and varies depending on the pulse amplitude, length, number of pulses and number of repeats [46].

Oxidation involves chains of complex chemical reactions which eventually lead to cell degradation and constant stimulation of their repair mechanisms [48]. Therefore, oxidation is a transient process. Consequently, it is challenging to detect and appropriately measure ROS and oxidation. In our study, the membrane oxidation was measured with the Click-iT^TM^ Lipid Peroxidation Imaging Kit. This reagent detects alkyne-containing modified proteins which are the products of oxidation. Thus, it enables the indirect quantification of cells’ oxidative stress. To measure the ROS content, we applied CM-H_2_DCFDA. The dye is cleaved by the intracellular esterase and remains trapped inside the cells, thus enabling the detection of intracellular ROS.

Yo-Pro uptake studies allowed us to determine the cell membrane permeability after the application of the IRE protocol on melanoma cells. Curiously, only after the application of 4 kV/cm PEF, the membrane permeability was significantly affected 30 min after the electroporation. Thus, due to the differences between Yo-Pro and trypan blue uptake, we might conclude that the 2 kV/cm pulses did not induce the formation of long-lived electropores during the experiments. Initially, during the EP, the cell membranes were permeabilized, but their permeability decreased at the end of the PEF treatment. Concerning IRE, after a 24 h incubation, the viability of melanoma decreased drastically as reported by low mitochondrial activity. The application of such high voltages leads to the reorganization of the membranes of the cells: the latter are completely disrupted, show drastic blebbing and release the cytoplasmic content to the extracellular space. Aside from the increased permeability of the membrane, the cells were shown to undergo voltage-dependent ROS bursts and lipid oxidation. Indeed, the effect of high-voltage PEFs on cell morphology is similar to the one elicited by exposure to H_2_O_2_ to induce oxidation [49]. Quite interestingly, lipid oxidation does not appear to be voltage-dependent in the 2 to 4 kV/cm range. However, the increase of the electric field from 2 to 4 kV/cm leads to a significantly higher ROS burst. In this report, therefore, the lipid oxidation correlates with the length of the pulses which is consistent with the results reported by Breton et al. [50]. In case the decrease of cell viability is the desired effect, the enhancement of cytotoxicity of PEFs can be achieved by higher oxygen content in the medium. Conversely, cell hypoxia can attenuate this effect [51]. Several studies describe the phenomenon that the oxidation of lipid membrane can enhance its permeabilization [52,53].

We analyzed the effects of PEFs of intensities below the EP threshold on the same melanoma cells to analyze the ROS generation and lipid membrane oxidation they give rise to. The lower intensity pulses (200–400 V/cm) do not seem to induce the permeability or long-term reduced viability of the cells. In the case of the short (0.1 ms) pulses, except slight lipid oxidation, the PEFs do not induce the electroporation or permeabilization of the membranes to the standards other researchers used. Indeed, neither loss of viability nor structural changes were observed in the cells in comparison to the control. The level of lipid oxidation measured was, however, a little higher. Unlike under the IRE standard protocols treatment, after the application of 200–400 V/cm (0.1 ms) pulses, the morphology of the cell remained unchanged.

In contrast, elongated pulses (10 ms) below the electroporation threshold led to substantial voltage-dependent lipid oxidation and voltage-independent ROS stress (in the range 200–400 V/cm). In contrast, the 600 V/cm, 10 ms pulses, membrane permeability after PEF treatment remained low. The long-term viability of the cells was also affected only when the 600 V/cm pulses were applied. Noticeably, the ultrastructure studies of the membrane system revealed that elongated pulses (400 V/cm, 10 ms) induced morphological changes in the membranes of the internal organelles (Figure 4).

From the above-mentioned data (summarized in Table 1), we conclude that the main factor affecting the cell’s viability is the integrity of the cell membrane. Conversely, changes in the intracellular membrane organelles do not lead to cell death but rather signals the inner-derived ROS burst. Moreover, cells characterized by the same ROS content and the same level of lipid oxidation react differently regarding the reversibility of the changes to the cell membrane.

In summary, given the fact that PEF treatments were considered to exert a high impact on the oxidation of the cells and therefore of their membranes’ lipids, the study aimed to describe the mechanism of lipid oxidation on melanoma cells following such treatment. Electrolysis is at the origin of the generation of extracellular ROS in the media which if important might lead to plasma membranes’ lipid oxidation. After the application of 0.1 ms pulses below the EP threshold, ROS content is not present in the cell, however, slight lipid peroxidation occurs. In microsecond IRE, which also uses 0.1 ms pulses, ROS are probably originating from the internal organelles, such as mitochondria or endoplasmic reticulum, as pure data show clearly intracellular membranes’ disruption after IRE PEFs. However, the electrolysis might also play a role in the generation of extracellular ROS.

**Figure 8:**
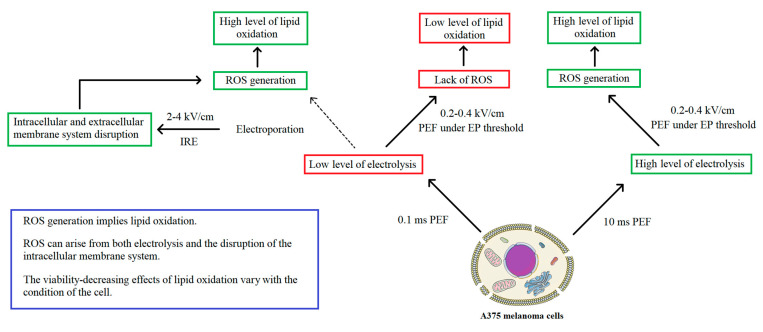
Mechanisms of ROS generation during PEF treatment.

In the case of the 400 V/cm, 10 ms pulses, the generation of ROS may arise from both extracellular excessive electrolysis and the disruption of the internal membranous organelles. In contrast, 200 V/cm, 10 ms PEF does not induce any changes in the membrane morphology of the cell, and therefore ROS are probably originating mostly from the extracellular compartment. Despite the high ROS content and lipid oxidation after treatment ith 10 ms pulses, PEF below the electroporation threshold (200–400 V/cm) has no impact on the viability of the cells both instantly after treatment with PEF and after 24 h of incubation. The detailed mechanism is presented in Figure 8. The combination of electrolysis and electroporation has been proposed as a promising approach [54]. It is based on the hypothesis that electrolysis products after permeabilizing the cells can enter the cell interior and trigger cell death. Therefore, the combination of pulses may also provide additional cytotoxicity enhancing the effect of standalone IRE or electrolysis.

## 4. Materials and Methods

### 4.1. Cell Culture

The melanotic melanoma A375 (ATCC^®^ CRL-1619™) cell line was obtained from the skin of a 54-year-old female patient. Cells were cultured as a monolayer in Dulbecco’s Modified Eagle’s Medium (DMEM, Sigma-Aldrich, St. Louis, MO, USA). The medium was supplemented with 10% fetal bovine serum (FBS, Sigma-Aldrich) and antibiotic (streptomycin/penicillin). The cells were incubated at 37 °C in a humidified atmosphere containing 5% CO_2_. When needed, the cells were washed with PBS and removed by trypsinization (0.025% trypsin and 0.02% EDTA; Sigma-Aldrich).

### 4.2. Electric Field Treatment

Electric field treatments were performed using the BTX 830 Electroporator (ECM830 Square Wave Electroporation System; BTX, Syngen Biotech, Wroclaw, Poland), calibrated before use. Electroporation cuvettes (VWR) with electrode gap 1 mm (BTX, Syngen Biotech, Poland) were used in the procedure. All protocols involved the use of 8 electric pulses. The experiments with standard IRE protocols involved the application of 2000 V/cm and 4000 V/cm, 0.1 ms pulses. PEFs below the EP threshold included 200 and 400 V/cm electric pulses of 0.1 ms duration. A 600 V/cm pulse was added to show the changes in membrane permeabilization. Finally, protocols using 10 ms pulses of intensities 200 and 400 V/cm were used.

The cells were detached from the culture flasks by trypsinization (0.025% trypsin and 0.02% EDTA; Sigma-Aldrich). In the count of 5 × 10^4^ cells per well, cells were suspended in a cuvette using a 10 mM phosphate buffer (pH = 7.4, 1 mM MgCl_2_ and 250 mM sucrose). Afterward, the samples were subject to the PEFs. After the procedure, the electroporation buffer was replaced with a culture medium (DMEM), and the cells were seeded on 96-well plates. The cell viability measurements (MTT) were carried out after 24 h of incubation.

### 4.3. Yo-Pro Uptake Studies

The permeabilization of the melanoma cells (A375) in response to PEF treatments was analyzed by flow cytometry (Cube-6, Sysmex EUROPE GmbH, Warsaw, Poland). The cells were detached from the culture flasks with trypsin, centrifuged and suspended in electroporation phosphate buffer (Na_2_HPO_4_/NaH_2_PO_4_, MgCl_2_, sucrose). Cells were maintained in suspension and pulsed in a cuvette (VWR) with two aluminum plate electrodes (1 mm gap). Afterward, the cells were treated with protocols described in paragraph 4.2. Then, the cells were incubated for 1 min. In the next step, cells were resuspended in 0.3 mL of PBS. Flow cytometry analysis was performed using a Cube 6 flow cytometer (Sysmex, Warsaw, Poland). The fluorescence of Yo-Pro was excited with a 488 nm laser and assessed with the FL-3 detector (700/50). Data were collected and analyzed by CyView software (Sysmex, Warsaw, Poland).

### 4.4. Trypan Blue Cells Staining

Permeabilization changes were assessed by trypan blue staining of the cells instantly and 30 min after the PEF treatments. The cells underwent electroporation with the protocol previously described. Instantly after PEF application, the first portion of the cells was stained with trypan blue by mixing cells in medium with trypan blue at a 3:1 ratio. The photographs were taken. The remaining cells’ suspension was carried under culture conditions for 30 min. After the time the cells were stained with trypan blue, and the photographs were taken. After the experiment, the percent of the stained cells was plotted.

### 4.5. MTT Viability Assay

The long-term viability of the cells was analyzed by mitochondrial activity assay. The culture medium was removed from each of the wells, and 100 μL of 0.5 mg/mL MTT (3-(4,5-dimethylthiazol-2-yl)-2,5-diphenyltetrazolium bromide, Sigma-Aldrich) solution in PBS buffer was added. After 2 h of incubation at 37 °C, acidified isopropanol (100 μL, 0.04 M HCl in 99.9% isopropanol) was added to dissolve the formazan crystals. The samples were fully dissolved by the pipet mixing technique. The absorbance of each well was measured at 570 nm using a multiplate reader (GloMax, Promega, Walldorf, Germany). The results were expressed as the percentage of viable cells relative to untreated control cells.

### 4.6. Confocal Microscopy Studies

CellMask Deep Red staining was performed to visualize the distribution and structure of the membranes and nuclei in A375 cells. After the electroporation experiment, the cells were incubated on cover glasses in Petri dishes overnight to attach. After that, 15 min incubation with CellMask Deep Red (1:1000; Thermo Fisher, C10046) was performed, then cells were fixed in 4% formalin solution. Fluorshield^TM^ with DAPI (4,6-diamidino-2-phenylindole) was applied to visualize the nuclei and to mount the cells. The samples were observed on the Olympus FluoView FV1000 confocal laser scanning microscope (Olympus, Tokyo, Japan).

### 4.7. Holotomographic Microscopy Studies

Live holographic tomography was performed using a 3D Cell Explorer microscope (Nanolive SA, Ecublens, Switzerland). Following the PEF treatments, the A375 cells were grown for 24 h and imaged using 35 mm Ibidi glass-bottom µ-Dish dishes (Ibidi GmbH, München, Bayern, Germany). Control samples—i.e., without PEF treatment—were analogously prepared. During imaging, a sufficient amount of CO_2_ was maintained by using a CO_2_-independent culture medium (Sigma-Aldrich). The temperature was set to 37 °C and controlled using an Ibidi Heating System (Ibidi GmbH, München, Bayern, Germany). Photographs of the samples were taken and analyzed.

### 4.8. ROS Burst Fluorescent Microscopy Studies

CM-H_2_DCFDA (C6827, Thermo Fisher Scientific, Waltham, MA, USA) was applied to assess the general oxidative stress after electroporation of A375 cells. The cells were PEF-treated and afterward incubated on cover glasses in Petri dishes for 12 h to attach. After that, 30 min incubation with CM-H_2_DCFDA was performed following the instructions of the manufacturer. Afterward, the cells were washed with PBS three times. The samples were observed on the Olympus BX53F2 microscope (40×, Olympus, Tokyo, Japan) after blue laser excitation (488 nm, Olympus, Tokyo, Japan). The fluorescence intensity of the samples was analyzed in the ImageJ software.

### 4.9. Lipid Oxidation Fluorescent Microscopy Studies

Click-iT^TM^ Lipid Peroxidation Imaging Kit—Alexa Fluor^TM^ 488 (C10446, Thermo Fisher Scientific, Waltham, MA, USA) was applied to assess the oxidation of the lipid membranes after electroporation of A375 cells. The cells were electroporated and seeded on the cover glasses in Petri dishes for 12 h to attach. After that, 24 h incubation with Click-iT^TM^ LAA compound was performed. Then, the cells were fixed with a formaldehyde solution, and the staining protocol of the manufacturer was performed. The samples were observed on the Olympus BX53F2 microscope (20×, Olympus, Tokyo, Japan) after blue laser excitation (488 nm, Olympus, Tokyo, Japan). The fluorescence intensity of the samples was analyzed in the ImageJ software.

### 4.10. Statistical Analysis

The experiments were performed in replicates. Data were expressed as mean ± SD and analyzed by one-way ANOVA (in GraphPad Prism 8), with *p* < 0.05 being considered statistically significant.

## 5. Conclusions

Microsecond pulsed electric field is an effective method for the induction of cell death among melanoma cells in the IRE protocol. By the decrease in cell viability, increase in the membrane permeability and the ultrastructural changes in the membranous system, the procedure could be used for inducing cell death in melanotic melanoma. The death of melanoma cells is specifically related to the changes in the organization of the cellular membranes and not solely to the generation of ROS nor lipid oxidation.

## Figures and Tables

**Table 1:** Effects of the different PEF protocols on the human melanotic A375 melanoma cells.

	Permeability of the Membrane during PEF Treatment	Permeability of the Membrane after PEF Treatment	Long-Term Reduced Viability	ROS Burst	Lipid Oxidation	Change in the Plasma Membrane Integrity	Changes in the Membranes of the Internal Organelles
**IRE protocols (2–4 kV/cm, 0.1 ms, 8 pulses).**	YES	HIGH	YES	YES, voltage-dependent	HIGH, voltage-independent	YES	YES
(**200–400 V/cm, 0.1 ms, 8 pulses).**	NO (same as control)	NO	NO	NO	LOW	NO	NO
**(200–400 V/cm, 10 ms, 8 pulses).**	NO (same as control)	LOW	NO	YES, voltage-independent	HIGH, voltage-dependent	NO	YES, 400 V/cm PEF exclusively

## Data Availability

The data presented in this study are available on request from the corresponding author.
